# Cost analysis of chronic heart failure management in Malaysia: A multi-centred retrospective study

**DOI:** 10.3389/fcvm.2022.971592

**Published:** 2022-11-02

**Authors:** Siew Chin Ong, Joo Zheng Low, Wing Yee Yew, Chia How Yen, Muhamad Ali S. K. Abdul Kader, Houng Bang Liew, Abdul Kahar Abdul Ghapar

**Affiliations:** ^1^Discipline of Social and Administrative Pharmacy, School of Pharmaceutical Sciences, Universiti Sains Malaysia, Pulau Pinag, Malaysia; ^2^Hospital Sultan Ismail Petra, Ministry of Health, Kelantan, Malaysia; ^3^Hospital Queen Elizabeth, Ministry of Health, Sabah, Malaysia; ^4^Institute for Clinical Research, National Institute of Health, Ministry of Health, Selangor, Malaysia; ^5^Clinical Research Centre Hospital Queen Elizabeth II, Ministry of Health, Sabah, Malaysia; ^6^Hospital Pulau Pinang, Ministry of Health, Pulau Pinang, Malaysia; ^7^Hospital Queen Elizabeth II, Ministry of Health, Sabah, Malaysia; ^8^Hospital Serdang, Ministry of Health, Selangor, Malaysia

**Keywords:** heart failure, cost analysis, economic burden, cost-of-illness, cost

## Abstract

**Background:**

Estimation of the economic burden of heart failure (HF) through a complete evaluation is essential for improved treatment planning in the future. This estimation also helps in reimbursement decisions for newer HF treatments. This study aims to estimate the cost of HF treatment in Malaysia from the Ministry of Health’s perspective.

**Materials and methods:**

A prevalence-based, bottom-up cost analysis study was conducted in three tertiary hospitals in Malaysia. Chronic HF patients who received treatment between 1 January 2016 and 31 December 2018 were included in the study. The direct cost of HF was estimated from the patients’ healthcare resource utilisation throughout a one-year follow-up period extracted from patients’ medical records. The total costs consisted of outpatient, hospitalisation, medications, laboratory tests and procedure costs, categorised according to ejection fraction (EF) and the New York Heart Association (NYHA) functional classification.

**Results:**

A total of 329 patients were included in the study. The mean ± standard deviation of total cost per HF patient per-year (PPPY) was USD 1,971 ± USD 1,255, of which inpatient cost accounted for 74.7% of the total cost. Medication costs (42.0%) and procedure cost (40.8%) contributed to the largest proportion of outpatient and inpatient costs. HF patients with preserved EF had the highest mean total cost of PPPY, at USD 2,410 ± USD 1,226. The mean cost PPPY of NYHA class II was USD 2,044 ± USD 1,528, the highest among all the functional classes. Patients with underlying coronary artery disease had the highest mean total cost, at USD 2,438 ± USD 1,456, compared to other comorbidities. HF patients receiving angiotensin-receptor neprilysin-inhibitor (ARNi) had significantly higher total cost of HF PPPY in comparison to patients without ARNi consumption (USD 2,439 vs. USD 1,933, *p* < 0.001). Hospitalisation, percutaneous coronary intervention, coronary angiogram, and comorbidities were the cost predictors of HF.

**Conclusion:**

Inpatient cost was the main driver of healthcare cost for HF. Efficient strategies for preventing HF-related hospitalisation and improving HF management may potentially reduce the healthcare cost for HF treatment in Malaysia.

## Introduction

Heart failure (HF) is a complicated clinical diagnosis resulting from structural and/or functional abnormalities. These abnormalities reduce cardiac output and/or increase in intra-cardiac pressures at rest or during exertion (1). Over 60 million people globally have HF (2), and this number will rise with age (3, 4). HF accounts for 6–10% of all acute medical admissions and is a significant cause of hospital readmissions in Malaysia (5). Although life expectancies of HF patients have substantially improved with the advancement of therapies, the myocardial damage is irreversible, resulting more people eventually live with HF (6).

The yearly global economic burden directly contributed by HF was estimated at $108 billion per year, whereby direct cost accounted for about 60% of the total cost of treating HF (7). In affluent nations, such as the USA and Europe, costs associated with HF accounts for 1% to 2% of the annual healthcare budget (8). A systematic analysis of HF cost-of-illness (COI) indicated that hospitalisation contributed 44-96% to the overall direct cost of HF (9). A registry-based COI study conducted in a hospital in East Malaysia found that the mean cost of HF per patient was about USD 6,017 and inpatient cost contributed to about 90% of the total cost (10). The reported cost from Malaysia was considerably lower than the cost reported in the USA and European countries (11).

The rising prevalence of HF in Malaysia (2) is also expected to have a significant impact on the total healthcare expenditure. The data on the cost of HF is scarce and are currently primarily limited to the high-income countries of Western Europe and North America (12). There is almost a complete lack of data from the middle to low-income countries, although they represent over 80% of the world’s population (7). Lately, newly emerged therapies, for example sodium-glucose cotransporter-2 inhibitor (SGLT2i) and angiotensin receptor neprilysin inhibitor (ARNI), have shown superior cardiovascular protective outcomes in HF patients compared to conventional treatments (13–15). However, besides therapy effectiveness, economic effectiveness is also a critical consideration in decision making for policymakers. Hence, there is a pressing need for the government and health organisations to estimate the costs attributable to HF. The estimates would aid the better planning of care management in a rapidly growing and ageing population. Therefore, the objective of this study was to estimate the cost of HF treatment in Malaysia from the Ministry of Health (MOH)’s perspective. The estimates were categorised according to different ejection fraction (EF) classifications, i.e., HF with reduced EF (HF*r*EF), HF with mildly reduced EF (HF*mr*EF) and HF with preserved EF (HF*p*EF) and the NYHA classification (NYHA class I-IV).

## Materials and methods

### Study design

The study was a non-interventional and retrospective study. Existing data were used from the medical records of patients diagnosed with HF from the cardiology departments of three tertiary hospitals in Malaysia. Data was extracted from the patients’ medical records from the electronic Hospital Information System (eHIS) or hard copy medical files. The study obtained ethical approval from the Medical Research and Ethics Committee (NMRR-20-2379-56411).

### Study sites

Adult patients with HF were identified from the database maintained by the Cardiology Departments at Serdang Hospital (SH), Penang General Hospital (PGH) and Hospital Queen Elizabeth II (HQE2). PGH is the largest tertiary centre in the Northern region of Malaysia, while HQE2 is the second largest tertiary centre in the whole of East Peninsular Malaysia. SH is also one of the famous cardiac centres in the central region of Malaysia. Furthermore, all the three tertiary hospitals selected in the study have designated HF clinics for specialised care management of HF patients not available in most of the other hospitals. The hospitals are among the cardiology centres with the highest number of HF patients treated in Malaysia. Furthermore, the selected centres have a complete database of their HF patients, which allowed specific identification of the treated HF patients for the study. Other centres did not have an established and detailed database of their HF patients and this challenges patient identification for the retrospective nature of this study.

### Study population

**FIGURE 1:**
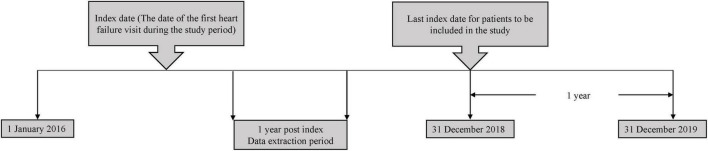
Study design.

The study population comprised of chronic adult HF patients aged ≥18 years old who received medical treatment in HF clinics at SH, PGH and HQE2 between 1 January 2016 to 31 December 2018. An index date corresponding to the date of the first HF-related visit at the hospitals (either in an outpatient or inpatient setting), between the study start and end date was recorded for each patient. Each patient was followed-up for up to 1 year from the index date (Figure 1). Patients who were referred to the corresponding HF clinics and diagnosed to have chronic HF based on clinical judgement by a physician, were included in the study. However, patients without details of their left ventricular ejection fraction (LVEF) and NYHA functional classes, diagnosed with cancer or participated in interventional clinical trial during the post-index period were excluded from the study.

All HF patients listed in the hospitals’ database were included in the current study. The estimated number of eligible patients were around 300-400 for the defined study period. The sample size was further confirmed by considering the precision (width of 95% confidence interval) and coefficient of variation (0.1) derived from the previous cost of illness study of HF in Malaysia (10), the minimum sample size for each groups was 62 patients based on the method published by Johnston *et al.* (16). Thus, the total sample size was 248 with four groups of NYHA classification. The total sample size was 320 after accounting for possible 30% of missing data. The current study included a total of 329 HF patients.

### Outcomes measured

The primary outcomes of this study were direct medical cost of HF and resource utilisation during the 12-month period following the index date. Out-of-pocket costs or self-paid services were not included in the current study. The healthcare cost of HF was defined as the cost of inpatient and outpatient consultation, interventional procedures, diagnostic tests and medications. The healthcare-related cost of HF and resource utilisation were calculated as per-patient-per-year (PPPY) and per-patient-per-event. Additionally, the cost of HF was categorised based on LVEF: HF*r*EF (with LVEF ≤ 40%), HF*mr*EF (with LVEF 41-49%) and HF*p*EF (LVEF ≥ 50%). In addition, costs of HF were segregated based on NYHA functional classes, underlying comorbidities, aetiologies of HF (ischaemic *vs.* non-ischaemic) and HF diagnosis during admission (primary *vs.* secondary). HF as the primary diagnosis was defined as hospitalisation with acute decompensated HF while HF as the secondary diagnosis was hospitalisation due to reasons other than acute decompensated HF. A history of coronary artery disease (CAD) or the presence of radiological evidence of coronary arteries stenosis indicated ischaemic aetiology and if otherwise, indicated as non-ischaemic aetiology.

### Cost calculation

An annual prevalence-based cost-of-illness approach was adopted from the perspective of the MOH, Malaysia. A bottom-up approach was used, whereby data on the quantity of healthcare resource utilisation were retrospectively extracted from patients’ medical records. The data included the number of outpatient visits, inpatient hospital stays due to HF, procedures and diagnostic performed, and medications utilised (Supplementary Tables 1–3). The annual cost of HF was the sum of annual inpatient and outpatient costs. The mean annual cost of inpatient setting was calculated as the total inpatient cost divided by total number of patients who had hospitalisation. Similar method was used to derived the mean annual cost of outpatient. Inpatient cost consisted of cardiac/general ward length of stay (LOS), intensive care unit LOS, coronary care unit LOS, medications, emergency department cost and the number of diagnostic tests and procedures performed or utilised during the hospital stay until discharge. The cost for a HF-related readmission within 30-days of first admission and subsequent hospitalisations were similarly calculated.

Outpatient cost consisted of total number of outpatient visits, medications and diagnostic tests performed. Any subsequent HF clinic visits (and subsequent HF-related resource usage) after the index date were taken into account to estimate the annual cost of HF. The cost of each component was computed by multiplying the unit cost by the quantity utilised for each component. Resources were converted to a 1-year cost based on the follow-up period. All measured resources were valued based on the MOH’s Full Paying Patient Tariff schedule of fees for a full-paying patient scheme provided by the Financial Department for the MOH hospitals (17). All citizens and legal residents of Malaysia have access to universal healthcare. The healthcare system is not paid by a national insurance scheme. Reimbursement systems only exist for private insurance companies which are not being covered by MOH. The government instead extensively subsidises the cost of care in the public institutions with revenues generated from taxation and other sources of funding. The full-paying fees paid by the non-citizen are the cost proxy best representing the unsubsidised charges for the healthcare services delivered in these non-for-profit MOH institutions. The charges listed were estimated based on the available cost information, inputs from the head of services and private fees survey (18). Thus, bottom-up methods capturing all the resources utilized and their associated costs in the MOH hospitals using full-paying tariff are the best representative of the cost from the MOH’s perspective. The unit cost of utilised drugs was valued based on the national price list of medicines provided by the Pharmacy Department’s Procurement Unit, using the hospital pharmacy’s average acquisition costs. Additionally, the cost of the percutaneous coronary intervention (PCI) was obtained from a published work in estimating the cost of PCI in five cardiac centres in Malaysia (19). All costs were converted to the 2021 values using the Malaysian consumer price index for the health domain (20). All costs were converted at the rate of RM 4.134/1 USD (21).

### Statistical analysis

The continuous variables were reported as mean [standard deviation (SD)] or median [interquartile range (IQR)] based on the distribution of data. The categorical variables were presented as numbers and percentages. Additionally, cost estimates were presented with a mean (SD) and median (IQR) where appropriate. Inferential statistics, including parametric [*t*-test, the analysis of variance (ANOVA)] and non-parametric (Mann-Whitney U test and Kruskal-Wallis H test) were employed to compare between groups. A generalised linear model (GLM) with log link and gamma distribution was used to determine the cost predictors for the annual cost of HF. The model was controlled for confounding variables, such as gender, age, ethnicity, underlying comorbidities, medications, health states (NYHA functional classes), LVEF and procedures. The cost ratios were reported for each variable with their respective 95% confidence interval (CI). Statistical significance was set at the value of p < 0.05. All analysis was conducted using Microsoft Excel^®^ (Microsoft, USA) and R (22).

## Results

### Demographic of study population

**FIGURE 2:**
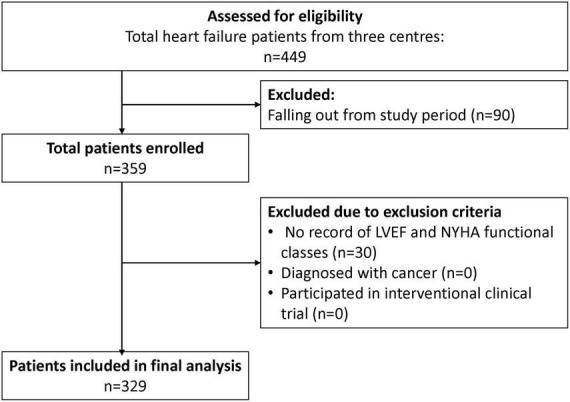
Patient inclusion and exclusion flow chart.

A total of 329 patients were included from the three study sites for the final analysis (Figure 2), with the majority of the study patients being males (82.1%) (Table 1). The HF patients were relatively young with a mean age of 54.6 years, SD 11.7 years. The Malay and Chinese ethnicities made up about 62% of the total study population. Majority of the HF patients were diagnosed with HF ≤ 1 year ago (71.4%). Comorbidities were highly prevalent among the HF patients with hypertension having the highest prevalence (68.4%). This was followed by coronary artery disease (56.2%), dyslipidaemia (47.1%) and type II diabetes mellitus (DM) (43.5%).

**TABLE 1:** Baseline demographic characteristics of heart failure patients.

Patient characteristics	Patients (*n* = 329)
**Gender, n (%)**	
Male	270 (82.1)
Female	59 (17.9)
Age (years), median (IQR); mean (SD)	55 (47-63); 54.6 (11.7)
**Ethnicity, n (%)**	
Malay	105 (31.9)
Chinese	98 (29.8)
Indian	50 (15.2)
^#^Others	76 (23.1)
**Duration of Heart Failure Diagnosis, n (%)**	
≤1 year	235 (71.4)
2–4 years	68 (20.7)
≥5year years	26 (7.9)
**Comorbidities, n (%)**	
Coronary artery disease	185 (56.2)
Hypertension	225 (68.4)
Type II diabetes mellitus	143 (43.5)
Dyslipidaemia	155 (47.1)
Chronic kidney disease	44 (13.4)
Atrial fibrillation	46 (14.0)
Cerebrovascular accident	23 (7.0)
Chronic obstruct pulmonary disease	12 (3.7)
Anaemia	7 (2.1)
Others	102 (31.0)
*eGFR (mL/min/1.73m^2^), median (quartiles)	38.0(22.5,47.1)
**Baseline EF, n (%)**	
HF*r*EF (≤40%)	263 (80.0)
HF*mr*EF (41–49%)	34 (10.3)
HF*p*EF (≥50%)	32 (9.7)
**Baseline NYHA Class, n (%)**	
Class I	154 (46.8)
Class II	128 (38.9)
Class III	32 (9.7)
Class IV	5 (1.5)
Unknown	10 (3.0)
**Aetiology of Heart Failure, n (%)**	
Ischaemic	203 (61.7)
Non-ischaemic	95 (28.9)
Unknown	31 (9.4)
**Centres, n (%)**	
Penang General Hospital	138 (41.9)
Serdang Hospital	77 (23.4)
Hospital Queen Elizabeth II	114 (34.7)
**Smoker status, n (%)**	
Current smoker	62 (18.8)
Ex-smoker	128 (38.9)
Not smoking	93 (28.3)
Unknown	46 (14.0)

EF, ejection fraction; eGFR, estimated glomerular filtration rate; HF*m*rEF, heart failure with mildly reduced ejection fraction; HF*p*EF, heart failure with preserved ejection fraction; HF*r*EF, heart failure with reduced ejection fraction; IQR, interquartile range; NYHA, New York Heart Association; SD, standard deviation.

*eGFR only available for patients with chronic kidney disease.

^#^Other ethnic included: Bajau, Dusun, Kadazan, Melanau, Murut and Iban.

Patients with HF*r*EF comprised of 80% of the total HF population in this study, but a significantly majority (p = 0.005) of them remained in a good NYHA functional class, i.e., class I (42.7%) and class II (44.3%). Ischaemic heart failure was the main aetiology of HF among the patients (61.7%).

### Resource utilisation of healthcare components

The mean (± SD) total cost of HF PPPY was USD 1,971 (± USD 1,255), while the median total cost of HF PPPY (IQR) was USD 581 (USD 304 – 989) (Table 2). The mean ± SD inpatient costs (USD 1,473 ± USD 1,790), contributed to about three-quarter of the mean total cost of HF PPPY. Medical procedures accounted for the highest percentage of cost PPPY (30.5%), followed by diagnostic tests (21.4%) and hospitalisation (21.4%). The mean cost of HF clinic visits and outpatient diagnostic tests contributed to about half of the mean outpatient cost. Another half of the mean of outpatient cost was contributed by outpatient medications.

**TABLE 2:** Health care resource utilisation and cost distribution of heart failure patients.

Setting	Cost component	Total utilisation, *n*	Utilisation PPPY, mean (SD)	Cost per PPPY, (USD) mean (SD) median (quartiles^a^)	% of cost PPPY^b^	Cost PPPY in each setting, (USD) mean (SD) median (quartiles^a^)	% of cost PPPY^b^
Outpatient *n* = 329	Clinic Visits	1,161	3.5 (2.3)	114 (76) 97 (65, 129)	5.8		25.3
	Medications (tablets/capsules/vials/ampoules)	991,517	3,014 (1,383)	209 (273) 103 (65, 202)	10.6	498 (348) 411 (238, 660)	
	Diagnostic Tests	2,150	6.5 (6.0)	174 (167) 123 (43, 266)	8.8		
Inpatient *n* = 101	Hospitalisations (bed-day)	522	5.2 (4.1)	422 (415) 305 (210, 445)	21.4	1,473 (1,790) 680 (517, 1,320)	74.7
	Medications (tablets/capsules/vials/ampoules)	5,712	56.5 (67.0)	29 (98) 4 (1, 19)	1.4		
	Diagnostic Tests	1,501	14.9 (13.2)	421 (297) 382 (215, 524)	21.4		
	Procedures	19	0.2 (0.4)	601(1505) 0 (0, 0)	30.5		
Total cost PPPY, (USD)							
Mean (SD)						1,971 (1,255)	100
Median (quartiles^a^)						581 (304, 989)	

PPPY, per patient per year; SD, standard deviation; USD, United States dollar.

Procedures included: 16 percutaneous coronary interventions, 2 pacemaker insertion, 1 implantable cardioverter defibrillator.

^a^First quartile and third quartile were used to describe the distribution of the cost because the costs were skewed to the right.

USD 1 = RM 4.134.

^b^Percentage of cost per patient per year was generated for mean cost only.

A total of 1161 visits to the HF clinics were made throughout the duration of follow up, with a mean of 3.5 visits PPPY. A total of 522 bed-days were recorded with 5.2 bed-days PPPY. Furthermore, patients who visited HF clinics consumed a higher number of medications of PPPY compared to hospitalised patients (3,014 *vs.* 56.5).

### Distribution of cost of heart failure based on left ventricular ejection fraction

The mean total costs (± SD) of HF PPPY for HF*r*EF, HF*mr*EF and HF*p*EF, were USD 1,909 (± USD 1,291), USD 2,471 (± USD 991), and USD 2,410 (± USD 1,226), respectively (Table 3). However, the median total cost of HF PPPY (IQR) for HF*r*EF was the highest USD 600 (USD 294 – 1,025), followed by HF*mr*EF USD 514 (USD 367– 837) and HF*p*EF USD 492 (USD 279 – 739) (p = 0.384).

**TABLE 3:** Cost distribution of heart failure patients categorised based on left ventricular function.

Setting	Cost component	HF*r*EF (EF ≤ 40%), *n* = 263		HF*mr*EF (EF 41–49%), *n* = 34		HF*p*EF (EF ≥ 50%), *n* = 32	
				
		Cost PPPY, (USD) mean (SD), median (quartiles^a^)	% of cost PPPY*^b^*	Cost PPPY, (USD) mean (SD), median (quartiles^a^)	% of cost PPPY *^b^*	Cost PPPY, (USD) mean (SD), median (quartiles^a^)	% of cost PPPY*^b^*
Outpatient, *n* = 329	Clinic Visits	116 (77)	6.1	110 (49)	4.4	107 (96)	4.4
		97 (65, 162)		97 (65, 129)		97 (65, 129)	
	Medications (tablets/capsules/vials/ampoules)	210 (280)	11.0	243 (281)	9.8	172 (201)	7.1
		102 (63, 182)		129 (77, 304)		100 (60, 185)	
	Diagnostic Tests	164 (157)	8.6	203 (175)	8.2	233 (223)	9.7
		121(43, 231)		174 (49, 301)		228 (32, 413)	
	Total	489 (353)	25.6	556 (333)	22.5	512 (326)	21.2
		395 (233, 644)		460 (353, 708)		445 (258, 634)	
Inpatient, *n* = 101	Hospitalisations (bed-day)	413 (415)	21.7	299 (160)	12.1	657 (519)	27.3
		305 (210, 445)		305 (235, 396)		541 (300, 717)	
	Medications (tablets/capsules/vials/ampoules)	20 (48)	1.0	13 (20)	0.5	178 (353)	7.4
		3 (1, 18)		5 (2, 12)		24(20, 85)	
	Diagnostic Tests	410 (271)	21.5	437 (121)	17.7	576 (649)	23.9
		379 (214, 519)		390 (351, 539)		330 (154, 664)	
	Procedures	577 (1,527)	30.2	1,166 (1,597)	47.2	486 (1,190)	20.2
		0 (0, 0)		0 (0, 2,915)		0 (0, 0)	
	Total	1,420 (1,795)	74.4	1,916 (1,686)	77.5	1,898 (1,985)	78.8
		662 (522, 1,206)		846 (818, 3,544)		983 (474, 2,962)	
Total cost PPPY, (USD)							
Mean (SD)		1,909 (1,291)	100	2,471 (991)	100	2,410 (1,226)	100
Median (quartiles^a^)		600 (294, 1,025)		514 (367, 837)		492 (279, 739)	

EF, ejection fraction; HF*mr*EF, heart failure with mildly reduced ejection fraction; HF*p*EF, heart failure with preserved ejection fraction; HF*r*EF, heart failure with reduced ejection fraction; PPPY, per patient per year; USD, United States dollar; SD, standard deviation.

^a^First quartile and third quartile were used to describe the distribution of the cost because the costs were skewed to the right.

^b^Percentage of cost per patient per year was generated for mean cost only.

USD 1 = RM 4.134.

The mean outpatient cost (± SD) for HF*r*EF was the lowest (USD 489 ± USD 353), but contributed to the highest proportion (25.6%) of the mean total cost of HF PPPY, among the three classes of LVEF. Additionally, the greatest cost contributor to the outpatient cost in patients with HF*r*EF was medication, which accounted for 42.9% of the total mean outpatient cost. The mean inpatient cost (± SD) for HF*r*EF was the lowest (USD 1,420 ± USD 1,795), followed by HF*mr*EF (USD 1,916 ± USD 1,686) and HF*p*EF (USD 1,898 ± USD 1,985). The largest contributor for mean inpatient cost in patients with HF*r*EF and HF*mr*EF, was the cost of procedures, which accounted for 40.6% and 60.9%, respectively.

### Annual cost of heart failure patients with underlying conditions

The median total cost of HF was the highest following the first year of HF diagnosis (USD 664). The median then decreased to USD 455 during the second to fourth year of diagnosis but bounced back to USD 464 (*p* = 0.022) during fifth year (Table 4). The mean total cost for HF PPPY (±SD) patients who had CAD as one of the comorbidities, was found to be the highest (USD 2,438 ± USD 1,456), in comparison to the other concurrent comorbidities. Additionally, HF patients who had type II diabetes mellitus (DM) as one of their underlying comorbidities, also had a significantly higher mean total cost PPPY than HF patients without type II DM (USD 2,406 *vs.* USD 1,597, *p* = 0.023).

**TABLE 4:** Annual cost of heart failure patients with different underlying conditions.

Conditions	*n* = 329	Cost PPPY (USD) mean (SD)	Cost PPPY (USD) median (quartiles^a^)
**Duration of HF diagnosis, n (%)**			
≤1 year	235 (71.4)	1,938(1,320)	664(350,1,038)
2–4 years	68 (20.7)	2,502(1,224)	455(257,732)
≥5year years	26 (7.9)	1,134(399)	464(367,708)
**Comorbidities, n (%)**			
Atrial fibrillation	46 (14.0)	1,543(807)	749(384,1,071)
Type II diabetes mellitus	143 (43.5)	2,406(1,444)	644(385,1,107)
Dyslipidaemia	155 (47.1)	2,238(1,466)	640(359,983)
Hypertension	225 (68.4)	1,893(1,150)	577(297,977)
Chronic kidney disease	44 (13.4)	2,417(1,512)	578(447,1,365)
Coronary artery disease	185 (56.2)	2,438(1,456)	568(373,1,022)
**Ejection fraction, n (%)**			
HF*r*EF (≤40%)	263 (80.0)	1,909(1,291)	600(294,1,025)
HF*mr*EF (41–49%)	34 (10.3)	2,471(991)	514(367,837)
HF*p*EF (≥50%)	32 (9.7)	2,410(1,226)	492(279,739)
**Baseline NYHA Class, n (%)**			
Class I	154 (46.8)	2,011(993)	476(276,925)
Class II	128 (38.9)	2,044(1,528)	662(398,996)
Class III	32 (9.7)	1,944(1,317)	801(461,1,537)
Class IV	5 (1.5)	1,762(870)	1,698(591,1,790)
**Aetiology of HF, n (%)**			
Ischaemic	203 (61.7)	2,475(1,478)	524(309,1,022)
Non-ischaemic	95 (28.9)	1,440(798)	714(354,1,009)
**Medication, n (%)**			
ARNi	22 (6.7)	2,439(1,301)	1,299(763,1,617)
ACEi^b^	224 (68.1)	1,942(1,280)	532(284,900)
ARB^c^	47 (14.3)	2,102(1,251)	748(395,1,212)
Beta-blocker^d^	312 (94.8)	1,940(1,265)	575(302,1,022)
MRA^e^	187 (70.8)	1,724(1,211)	607(304,1,030)
Combination^f^	157 (59.5)	1,830(1,289)	600(288,1,092)
**Centres, n (%)**			
Penang General Hospital	138 (41.9)	2,072(1,393)	720(406,1,271)
Serdang Hospital	77 (23.4)	2,800(1,587)	599(411,1,164)
Hospital Queen Elizabeth II	114 (34.7)	1,161(558)	441(237,775)

ACEi, angiotensin-converting enzyme inhibitors; ARB, angiotensin-receptor blockers; ARNi, angiotensin receptor-neprilysin inhibitor; HF, heart failure; HF*mr*EF, heart failure with mildly reduced ejection fraction; HF*p*EF, heart failure with preserved ejection fraction; HF*r*EF, heart failure with reduced ejection fraction; MRA, Mineralocorticoid receptor antagonist; NYHA, New York Heart Association; PPPY, per patient per year; RAAS, Renin-angiotensin-aldosterone system; SD, standard deviation; USD, United States dollar.

^a^First quartile and third quartile were used to describe the distribution of the cost because the costs were skewed to the right.

^b^ACEi included in the analysis were perindopril, ramipril and enalapril.

^c^ARB included in the analysis were losartan, telmisartan, irbesartan and valsartan.

^d^Beta-blocker in the analysis were bisoprolol and carvedilol.

^e^MRA in the analysis were spironolactone and eplerenone in HF*r*EF patients only.

^f^Combination therapy of RAAS inhibitors, beta-blocker and mineralocorticoid receptor antagonist in HF*r*EF patients only.

USD 1 = RM 4.134.

The median total cost of HF PPPY increased with severity of NYHA functional class. NYHA class I had the lowest cost of USD 476, while the most severe NYHA of class IV had the highest cost of USD 1,698. Differences in the cost among the functional classes were statistically significant (*p* = 0.006). HF patients who were concurrently treated with angiotensin receptor-neprilysin inhibitor (ARNi) had the highest mean ± SD total cost of HF PPPY (USD 2,439 ± USD 1,301). This data was significantly higher than HF patients not taking ARNi (USD 1,933 ± USD 1,245), *p* < 0.001. Conversely, HF patients on angiotensin-converting enzyme inhibitor (ACEi) were associated with lower mean total cost of HF PPPY, compared to patients not on ACEi (USD 1,942 *vs.* USD 2,034, *p* = 0.011). A similar trend was observed in HF*r*EF patients treated with a combination of renin-angiotensin-aldosterone system (RAAS) inhibitors, beta-blocker and mineralocorticoid receptor antagonist (MRA). These patients had a lower mean total cost of HF PPPY compared to the patients who did not receive this combination (USD 1,830 *vs.* USD 2,009, *p* = 0.530). The healthcare costs of HF for patients who received treatment in HQE2 (USD 1,161 ± USD 558) were significantly lower than patients followed-up in PGH (USD 2,072 ± USD 1,393) and SH (USD 2,800 ± USD 1,587), *p* < 0.001.

### Demographic and cost of patients who had hospitalisation events

Only 101 patients (30.7%) had at least one hospitalisation event(s) throughout the one-year duration of follow up from the index date (Table 5). There were no significant differences in the age and proportion of underlying comorbidities between HF patients who had hospitalisation and without hospitalisation. However, HF patients who had hospitalisation, had a significantly higher proportion of HF*r*EF (89.1% *vs.* 80.2%, *p* = 0.012), NYHA class II (45.5% *vs.* 38.9%, *p* < 0.001), NYHA class III (16.8% *vs.* 9.7%, *p* < 0.001) and non-ischaemic HF (38.6% *vs.* 28.9%, *p* = 0.038) in comparison to the overall study population. In addition, patients with a HF diagnosis of a duration of ≤ 1 year, were 2.1 times (*p* = 0.009) more likely to be hospitalised than those with a greater diagnosis of more than 2 years. Hospitalisation directly due to HF (primary diagnosis) accounted for 59.4% of all total admission. Hospitalised patients reported a 5.9% prevalence of 30-days HF related readmission. In addition, for patients who had previous hospitalisations, 12.9% of them had at least another hospitalisation episode within one year of follow up.

**TABLE 5:** Sub-analysis in patients who had heart failure hospitalisation.

Patient characteristics	Patients (*n* = 101)
**Gender, n (%)**	
Male	84 (83.2)
Female	17 (16.8)
Age (years), mean (SD)	53.9 (11.1)
**Ethnicity, n (%)**	
Malay	38 (37.6)
Chinese	29 (28.7)
Indian	17 (16.8)
Others	17 (16.8)
**Duration of Heart Failure Diagnosis, n (%)**	
≤1 year	82 (81.2)
2–4 years	14 (13.9)
≥5 year	5 (4.9)
**Comorbidities, n (%)**	
Coronary artery disease	52 (51.5)
Hypertension	71 (70.3)
Type II diabetes mellitus	47 (46.5)
Dyslipidaemia	48 (47.5)
Chronic kidney disease	15 (14.9)
Atrial fibrillation	16 (15.8)
Cerebrovascular accident	7 (6.9)
Chronic obstructive pulmonary disease	5 (5.0)
Anaemia	3 (3.0)
Others	27 (26.7)
*eGFR (mL/min/1.73m^2^), median (quartiles^a^)	38 (21.3, 40)
**Baseline EF, n (%)**	
HF*r*EF (≤40%)	90 (89.1)
HF*mr*EF (41–49%)	5 (5.0)
HF*p*EF (≥50%)	6 (5.9)
**Baseline NYHA Class, n (%)**	
Class I	31 (30.7)
Class II	46 (45.5)
Class III	17 (16.8)
Class IV	3 (3.0)
Unknown	4 (4.0)
**Aetiology of Heart Failure, n (%)**	
Ischaemic	54 (53.5)
Non-ischaemic	39 (38.6)
Unknown	8 (7.9)
**HF diagnosis at admission, n (%)**	
Primary	60 (59.4)
Secondary	41 (40.6)
Duration of hospitalisation (days), mean (SD)	5.2 (4.1)
Prevalence of 30 days HF-related readmission, n (%)	6 (5.9)
Prevalence of ≥2 HF-related admission per year, n (%)	13 (12.9)
**Total cost of HF PPPY (USD)**	
Mean (SD)	1,997 (1,826)
Median (Quartiles^a^)	1,297 (901, 2,267)
**Total cost of HF PPPY based on frequency of hospitalisation per year (USD)**	
Single HF-related hospitalisation per year	
Mean (SD)	1,770 (1,134)
Median (Quartiles^a^)	544 (290, 950)
**≥2 HF-related hospitalisations per year**	
Mean (SD)	3,320 (1,707)
Median (Quartiles^a^)	3,913 (1,698, 4,552)
**Cost of HF-related hospitalisation per admission (USD)**	
Mean (SD)	1,269 (1,638)
Median (Quartiles^a^)	652 (477, 1,178)
**Cost of HF-related hospitalisation per admission based on types of wards**	
General cardiology ward	
Mean (SD)	1,070 (1,460)
Median (Quartiles^a^)	603 (470, 896)
**Coronary care unit**	
Mean (SD)	2,411 (2,142)
Median (Quartiles^a^)	1,320 (813, 3,692)
**Cost of 30 days HF-related readmission per admission (USD)**	
Mean (SD)	485 (149)
Median (Quartiles^a^)	457 (395, 567)
**Cost of HF-related readmission within 1 year (USD)**	
Mean (SD)	1,196 (1,407)
Median (Quartiles^a^)	492 (386, 717)
**Cost of HF-related hospitalisation per admission based on HF diagnosis on admission (USD)**	
**Primary**	
Mean (SD)	904 (1,146)
Median (Quartiles^a^)	598 (465, 857)
**Secondary**	
**Overall**	
Mean (SD)	1,803 (2,068)
Median (Quartiles^a^)	755 (574, 2,570)
**Acute coronary syndrome**	
Mean (SD)	1,650 (1,477)
Median (Quartiles^a^)	777 (572, 2,655)
**ICD implantation^b^**	
Mean (SD)	11,252 (NA)
Median (Quartiles^a^)	11,252 (NA)
**Atrial fibrillation**	
Mean (SD)	827 (506)
Median (Quartiles^a^)	702 (577, 952)

EF, ejection fraction; eGFR, estimated glomerular filtration rate; HF, heart failure; HF*m*rEF, heart failure with mildly reduced ejection fraction; HF*p*EF, heart failure with preserved ejection fraction; HF*r*EF, heart failure with reduced ejection fraction; ICD, implantable cardioverter-defibrillator; NA, not applicable; NA, not applicable; NYHA, New York Heart Association; PPPY, per patient per year; USD, United States dollar; SD, standard deviation.

^a^First quartile and third quartile were used to describe the distribution of the cost because the costs were skewed to the right.

^b^SD and interquartile range cannot be generated for ICD implantation because of single admission was recorded.

*eGFR only available for patients with chronic kidney disease. USD 1 = RM 4.134.

The mean total cost (± SD) of HF PPPY for patients who had HF-related hospitalisation was four times higher than patients without hospitalisation USD 1,997 (± USD 1,826) *vs.* USD 486 (± USD 321), (*p* < 0.001). In addition, the mean total cost (± SD) of patients with multiple HF-related hospitalisation per year was significantly higher than those with single HF-related hospitalisation per year (USD 3,320 *vs.* USD 1,770, *p* < 0.001). The mean cost of HF-related hospitalisation (± SD) for each admission was USD 1,269 (± USD 1,638). The mean cost of HF-related hospitalisation (± SD) in patients who experienced coronary care unit (CCU) admission was significantly higher than those admitted to general cardiac ward only (USD 2,411 *vs.* USD 1,070, *p* = 0.033). Furthermore, the mean total cost was lower when HF was the primary diagnosis on admission, compared to when HF was a secondary diagnosis (USD 904 *vs.* USD 1,803, *p* = 0.005).

### Determination of cost predictors

Table 6 shows the ten variables that were significant predictors for the mean total cost of HF PPPY. HF patients from the indigenous ethnicity (others) had significantly lower mean total cost of HF PPPY by 31% (95% CI 0.55 – 0.87, *p* = 0.001), compared to patients of the Malay ethnicity. Moreover, HF patients on ACEi as part of their treatment regimen had a significantly lower mean of total cost of HF PPPY by 21% (95% CI 0.66 – 0.93, *p* = 0.006), as compared to patients who did not receive ACEi. Patients with hypertension had a significantly lower total mean cost than those without hypertension (cost ratio: 0.83, 95% CI 0.70–0.98, *p* = 0.027).

**TABLE 6:** Predictors of mean annual cost of heart failure.

Variables	Cost ratio	95% Confidence interval	*P*-value
**Model 1**			

**Demographics**			
Female	*Reference*		
Male	1.06	0.87 – 1.28	0.571
Age ≥ 65 years	0.87	0.72 – 1.06	0.159
Malay	*Reference*	–	−
Chinese	0.87	0.72 – 1.06	0.162
Indian	0.81	0.64 – 1.02	0.068
Others	0.69	0.55 – 0.87	0.001*
**Duration of heart failure diagnosis**			
≤1 year	*Reference*		
2–4 years	0.97	0.80 – 1.17	0.740
≥5year years	1.00	0.76 – 1.33	0.993
**Comorbidities**			
Hypertension	0.83	0.70 – 0.98	0.027*
Type II diabetes mellitus	1.20	1.02 – 1.41	0.023*
Dyslipidaemia	1.20	1.03 – 1.40	0.021*
Chronic kidney disease	1.33	1.07 – 1.66	0.011*
Atrial fibrillation	1.26	1.01 – 1.58	0.047*
Cerebrovascular accident	0.99	0.74 – 1.35	0.931
Chronic obstructive pulmonary disease	1.22	0.82 – 1.90	0.340
Anaemia	0.96	0.58 – 1.69	0.880
**Left ventricular ejection fraction**			
HFpEF (≥50%)	*Reference*		
HFmrEF (41–49%)	0.91	0.65 – 1.27	0.572
HFrEF (≤40%)	0.87	0.67 – 1.11	0.276
**NYHA**			
Class I	*Reference*	–	−
Class II	1.04	0.88 – 1.24	0.641
Class III	0.92	0.70 – 1.22	0.546
Class IV	1.47	0.84 – 2.83	0.214
HF Hospitalisation	2.78	2.31– 3.36	< 0.001*
Percutaneous coronary intervention	2.87	1.98 – 4.26	< 0.001*
Coronary angiogram	1.33	1.11 – 1.60	0.003*
ARNi	2.27	1.70 – 3.10	< 0.001*

**Model 2**			

ACEi	0.79	0.66 – 0.93	0.006*

ACEi, angiotensin converting enzyme inhibitors; ARNi, Angiotensin receptor-neprilysin inhibitor; HF*m*rEF, heart failure with mildly reduced ejection fraction; HF*p*EF, heart failure with preserved ejection fraction; HF*r*EF, heart failure with reduced ejection fraction; NYHA, New York Heart Association.

*Indicated significant difference of p-value < 0.05, assessed using multivariate generalised linear model (link = logarithm, distribution = gamma).

–Coronary artery disease was not included in the model as coronary angiogram mediated the mean annual cost of heart failures.

–ARNi was removed in the model 2 due to collinearity with ACEi, which removed the true effect of ACEi on the cost of heart failure. Replacing ARNi with ACEi did not change the model  fit and level of significance of other variables on the cost of heart failure.

–Assumptions of generalised linear model were checked and met.

Conversely, HF patients with comorbidities, such type II DM and chronic kidney disease (CKD) significantly increased the mean total cost of HF PPPY by 20% (95% CI 1.02 – 1.41, *p* = 0.023) and 33% (95% CI 1.07 – 1.66, *p* = 0.011), respectively. Patients with AF had significantly higher total mean cost than those without AF (cost ratio: 1.26, 95% CI 1.01 – 1.58, *p* = 0.047). Dyslipidaemia also significantly increased the total mean cost by 20% (95% CI 1.03 – 1.40, *p* = 0.021). Furthermore, patients who had HF-related hospitalisation were significantly associated with 2.78 times (95% CI 2.31 – 3.36, *p* < 0.001) higher mean total cost of HF PPPY, compared to non-hospitalised HF patients. Moreover, HF patients that underwent medical procedures, such as PCI (cost ratio 2.87, 95% CI 1.98 – 4.26, *p* < 0.001) and coronary angiogram (cost ratio 1.33, 95% CI 1.11 – 1.60, *p* = 0.003) were found to have significantly higher mean total cost of HF PPPY. In addition, HF patients that received ARNi treatment had a mean total cost HF PPPY of 2.27 times (95% CI 1.70 – 3.10, *p* < 0.001) higher than patients who did not receive ARNi treatment.

## Discussion

### Demographic of study population

Heart failure (HF) is a global public health challenge and also affects Malaysians. The present study is the first to assess healthcare costs and resource utilisation among HF patients categorised based on EF and NYHA classification of three major tertiary hospitals located in West and East Malaysia. The study’s findings have provided evidence on the economic burden of HF toward Malaysia’s healthcare system.

The mean age of the study population was 54.6 years, which was similar to the population of HF-related studies previously done in Malaysia (10, 23, 24). However, the cost-of-illness (COI) analyses done in Asian countries like Korea (25), Japan (26), and China (27), found that the mean age of their study population was older than our Malaysian population (range: 66 – 81 years). Furthermore, the mean age of the study population of COI done in western countries (range: 71 – 77 years), were also older than the Malaysian HF studies (28–30). In the present study, the most dominant HF phenotype was HF*r*EF. Studies found that HF*r*EF are more commonly found among younger patients (31, 32), having CAD as the aetiology of HF (4, 32) and among men (32). Observational studies in Malaysia found that 40-60% of HF cases were caused by CAD (5, 23, 33).In addition, the prevalence of the traditional risk factors for CAD, such as diabetes, hypertension, and dyslipidaemia, has increased steadily among Malaysia’s population since 2011 (34), translated into a higher risk of developing myocardial infarction at a younger age and eventually leading to HF*r*EF (31, 32). These findings shed some light on the age of HF patients in Malaysia, who were diagnosed at a much younger age compared to patients from other Asian and western countries. On the other hand, a high proportion of HF patients in the study were male patients. This finding was supported by a COI study done by Shafie et al., which had 85.9% of male patients (10). Additionally, the Malaysia Heart Failure Registry (MyHF) consisting of a total of 2673 patients reported that about two-thirds of the HF patients were males, and 75% of the patients with HF*r*EF were males (24).

In the present study, we found that the prevalence of patients with HF*p*EF (9.7%) was lower than in studies from the European Society of Cardiology (ESC) HF long-term registry (16.0%) (35), China (43.0%) (36) and Japan (61.9%) (37). Patients in the present study developed HF at a much younger age than patients in the studies mentioned above (ESC registry: 68.6 years; China: 71.3 years; Japan: 71.7 years), and HF*p*EF patients are more likely to be older (32). In addition, HF*p*EF was less likely to have ischaemic aetiology (32), but 61.7% of patients in the present study had ischaemic aetiology. Furthermore, a cost study involving two major cardiac centres in Malaysia also reported a lower prevalence of HF*p*EF (5.9%), and the dominant HF aetiology was CAD (52.9%) (38). Differences in the demographic characteristics and aetiologies between studies from different nations explained variation in the distribution of HF phenotypes.

### Comparison of total cost of heart failure per heart failure patient per-year

A systematic review that analysed 35 COI studies reported that the annual health care cost related to HF per patient was between USD 1,042 and USD 96,918 (10). Another systematic review that only included COI studies done in the USA showed that the mean cost of HF PPPY was USD 30,940, but the cost increased greatly to USD 53,601 when patients had specific comorbidities (39). The mean total cost of HF in our study was USD 1,971, which is lower compared to other countries (39–45). In East Asian countries, the COI studies related to HF done in China (USD 4,820) (27) and Japan (USD 12,490) (26) reported a higher cost than our study, with the exception of one study done in Korea (3), which reported their annual cost per patient at USD 977.

A COI study with a relatively small sample size that studied only stage C HF patients in Malaysia, found that the annual cost of HF per patient was USD 6,017 (10). The estimated mean HF cost was higher than the mean cost reported in the present study because the patients in the former study underwent more procedures than patients in this study (0.6 *vs.* 0.2 PPPY) (10). In addition, the previous study only recruited patients who had at least one episode of decompensated HF in the past six months that required hospitalization which cost higher relatively to the current study. A cost study which estimated the cost of HF in four Asian countries reported that the cost of HF in Malaysia was USD 1,820, which is congruent with our finding (USD 1,971). The estimated inpatient cost of HF generated from the Malaysian Disease Related Group (DRG) database was between USD 987 and 1,270, similar to the inpatient cost reported in this study (i.e., USD 1,473). The lower bound of the cost estimated from the Malaysian DRG database was from the primary hospitals where these facilities do not have in-house cardiologists, therefore complicated and expensive procedures could not be performed.

The wide variation in the costs reported across different countries may be mainly attributed to the relative prices of health care resources utilised to treat HF, treatment protocol, and most importantly, the methodology used in calculating the costs. Additionally, the differences could also be explained by the vast discrepancy in resource allocation by each country for their healthcare system in managing HF (7). Although the differences in the COI of HF were large, the data confirmed that HF is a massive economic burden on the healthcare system in each country. In addition, the use of local data to estimate the local economic burden of HF is crucial for decision making in the prioritisation of investments. The use of data from other countries could lead to an inaccurate estimation of the actual burden of HF.

### Resource utilisation by healthcare components

This study found that the inpatient cost was USD 1,473, while the outpatient cost was USD 498, which contributed 74.7% and 25.3% of the mean total cost of HF PPPY, respectively. A systematic review by Lesyuk *et al.* found that inpatient admission cost accounted for about 44% to 96% of the overall direct cost of HF (9). Annual inpatient cost was also the main cost driver of HF in the current study, which was in line with other studies (19, 29, 40, 46).

A breakdown from the mean total cost of HF in this study showed that the cost of procedures, such as PCI and implantation of anti-arrhythmic devices contributed the most significant proportion (30.5%). This was followed by the cost of diagnostic tests (30.2%), cost of hospital stays (21.4%), medications (12.0%) and cost of clinic visits (5.8%). Revascularisation procedures such as PCI and insertion of anti-arrhythmic devices are some of the expensive interventional procedures, which incur higher inpatient costs (47, 48). Two previous studies reported that these procedures contributed to about 22 - 40% of inpatient cost (49, 50), which was congruent to the findings in the present study.

Analysis of the individual components of HF care cost revealed that interventional procedures were the main cost driver of HF. These components showed the same outcome from another local study (10), as well as a study from another country (51). On the other hand, the mean hospitalisation cost in the current study was much lower than another local COI study (USD 1,473 *vs.* USD 5,454), although both studies had similar lengths of hospital stays (10). However, when comparing the median costs, which is more preferable due to exclusion of possible outliers, both studies reported comparable inpatient costs.

Previous studies reported that the cost of laboratory investigations and diagnostic tests ranged between 13 and 17% (10, 46, 52, 53). The relatively high diagnostic tests costs reported (30.2%) in our study, could be due to that majority of the patients (71.4%) were diagnosed with HF for less than 1 year. The high utilisation of diagnostic tests is part of the workup protocol for the diagnosis of HF. Other observational studies also reported that healthcare cost substantially increased during the first year of HF diagnosis, compared to the year prior to HF diagnosis (25, 29). Furthermore, the utilisation of diagnostic tests in the inpatient setting was higher than in the outpatient setting, and this was also observed in other studies (10, 44). Generally, hospitalised patients are more ill, and therefore require more frequent and various types of diagnostic tests for disease management and monitoring.

Our study found that the cost of medication was 12.0% of the mean total cost of HF PPPY. Previous report show that the cost of medication can range from 3 to 18% of the total healthcare cost of HF (10, 45, 54, 55). The variation in the cost of medication was partly contributed by the different treatment strategies adopted by the different institutions for the management of HF. Moreover, new pharmacological classes of therapy such as ARNi, was shown to reduce the risk of HF-related hospitalisation and cardiovascular mortality during trials (14). The clinical use of this therapy could have inflated the cost of medications, as these new patented medications are more expensive than generic medications (56).

### Variation in the cost of heart failure in patients with different underlying conditions

The complete trajectory of the lifetime cost of HF was the highest following the year of HF diagnosis. Previous studies have found that the healthcare cost of HF in newly diagnosed HF patients was higher than the prevalent group (25, 57, 58). The cost then decreased to a stable and relatively lower cost and surged again at the end of a patient’s life (29). Our study found that the median total cost of HF followed the trajectory described by Dunlay et al. (29). Patients from the current study had the highest cost incurred during the first year following HF diagnosis. The cost then decreased between 2 and 4 years and increased again after five years of HF diagnosis. The higher healthcare cost of HF during the first year of HF diagnosis was associated to a higher frequency of hospitalisation events (25). The current analysis of data found that patients who had been diagnosed with HF ≤ 1 year earlier had twice statistically significant more the odds of being hospitalised compared to patients who had been diagnosed with HF for ≥ 2 years, p = 0.034.

The present study also found that HF patients with CAD, had the highest mean total cost among all patients with comorbidities. Previous publications had also reported that HF patients concurrently diagnosed with CAD, were associated with a higher total healthcare cost of HF than those who did not have CAD (59, 60). HF patients with CAD incur higher costs because of receiving PCI and coronary artery bypass grafting (CABG), which are very expensive.

The present study found that patients with LVEF > 40% (HF*p*EF) had a higher total healthcare cost of HF compared to patients with LVEF ≤ 40% (HF*r*EF). This is because a significantly higher proportion of patients with HF*p*EF (80%, *p* = 0.041) are hospitalised with HF as their secondary diagnosis. A previous study reported that patients with LVEF ≥ 50% had an increased frequency of comorbidities, which could lead to extended hospitalisation and thus increased the total cost of HF (58). However, similarly with the current study, the differences among groups were not statistically significant after taking into consideration underlying comorbidities (58). Conversely, a Korean study that used a different definition for the categorisation of LVEF (≤35% *vs.*>35%) reported that the total healthcare cost of HF in patients with LVEF ≤ 35% was 2.5 times significantly higher than patients with LVEF > 35% (25). Similarly, another COI study showed that patients with LVEF ≤ 40% incurred a higher cost of hospitalisation. This was due to extended hospital stays and higher healthcare resource utilisation compared to patients with LVEF > 40% (61). However, the present study found that the mean duration of hospitalisation in patients with HF*p*EF was the longest (6.8 days), compared to HF*r*EF (5.1 days) and HF*mr*EF (3.6 days).

### Analysis of cost of patient with heart failure-related hospitalisation

The mean cost of HF-related hospitalisation per admission was USD 1,269. A systematic COI study in the USA found that the cost of hospitalisation due to HF per admission ranged between USD 7,777 and 32,381 (39). However, the cost per admission due to HF was lower in the Asian countries, ranging between USD 2,055 and 13,932 (3, 25, 27, 45).

A COI study that included over 20,000 hospitalisations to examine the cost of HF-related hospitalisations found that hospitalisation with HF as a secondary diagnosis was about USD 5,000 higher than those admitted with HF as the primary diagnosis (59). According to the study, among patients admitted with HF as the secondary diagnosis, the cost of hospitalisation was much higher with ischaemic heart disease as the primary diagnosis when compared to the primary diagnosis of non-pulmonary and non-cardiovascular conditions. Similarly, the present study observed that hospitalisation with HF as the secondary diagnosis incurred a higher cost at USD 899 than hospitalisation with HF as the primary diagnosis. About 88% of hospitalisation with HF as the secondary diagnosis in the current study was related to acute coronary syndrome. Compared to HF as the primary diagnosis for admission, there was a significantly higher proportion of patients admitted with HF as the secondary diagnosis, who received PCI (26.8% *vs.* 8.3%, *p* = 0.012) and coronary angiogram (68.3% *vs.* 45.0%, *p* = 0.021). This group contributed to the relatively higher cost of HF.

Heart failure (HF) is known to be an ambulatory care sensitive condition which leads to worsening conditions, and for which proper medical therapy in the outpatient settings can prevent disease progression (62). Additionally, low utilisation of guideline-recommended therapy in HF patients was associated with worsening HF condition (63). Addressing the factors that could improve outcomes after HF exacerbation would eventually decrease the expensive direct medical and healthcare resource utilisation costs (28). Furthermore, optimal care in the outpatient setting with timely intervention to prevent disease progression and complications could reduce hospitalisation events. This would ultimately help in shifting the cost of inpatient care to outpatient care, and thus create a cost-saving opportunity (10).

### Cost predictors for heart failure

The present study demonstrated that the mean total cost of HF PPPY was significantly higher when HF patients received ARNi as part of their pharmacological treatment. There was no statistical association between the NYHA functional classification and the use of ARNi, p = 0.8396. A cost-utility analysis was done on the treatment of sacubitril-valsartan in patients with HF*r*EF in the South East Asia region, separately by Thailand (64) and Singapore (65). It was found that the medication was not cost-effective based on their current cost-effectiveness threshold (CET). Although the therapeutic effects of sacubitril-valsartan were remarkable compared to enalapril in the trial (14), its low utilisation in real-world clinical practice was due to its high cost (64). Conversely, the budget impact analysis of introducing sacubitril-valsartan in the treatment strategy in France reported a potential budget-saving approach, due to avoidance of HF-related hospitalisation (66).

Patients who received ACEi as part of their HF treatment had significantly lower costs compared to patients who did not consume ACEi. A similar finding was also observed in one COI study in Korea (25). The potential cost saving of ACEi in HF patients of the current study was due to the off-patent nature of the ACEi used. Additionally, patients concurrently receiving a combination of ACEi, beta-blocker, and MRA had lower healthcare costs of HF. This is supported by the data that combination therapy with ACEi/ARB and beta-blockers resulted in a greater reduction in hazard of all-cause hospitalisation or mortality than monotherapy with either ACEi/ARB or beta-blocker (67). Besides, patients from the indigenous ethnicity (others) had a lower healthcare cost of HF than other ethnicities because they live in the interior and remote areas of East Malaysia and have low accessibility to medical treatment (68). They will need to travel quite a distance to the tertiary centre to seek medical treatment. In addition, the low accessibility to medical treatment in this population explained that the 30-day HF readmission rate was the lowest among all ethnicities in a study conducted in Malaysia (69). Furthermore, HF patients from the indigenous ethnicity had fewer PCI done (0 *vs.* 43.8%, *p* = 0.001) and received lesser ARNi treatment (0 *vs.* 45.5%, *p* = 0.02), than Malay patients. Moreover, the utilisation of traditional medicine was prevalent among this population (70). These explanations justified the lower healthcare cost of HF incurred by patients followed up in HQE2 because two-thirds of patients from HQE2 were of indigenous ethnicity. The costs of medication, diagnostic tests, and procedures in HQE2 were lower than in PGH and SH (Supplementary Table 4). The heterogeneity in the management of HF could explain the difference in the cost of HF across different centres in Malaysia. Patients with comorbidities such as DM (25, 29, 71, 72) and chronic kidney disease (CKD) (73) were associated with a higher cost of HF. HF patients with DM often had worse clinical outcomes compared to those without DM. Observational studies had consistently shown that the risk of hospitalisation was higher with patients who had DM (74–78). The present study reported that HF patients with DM had significantly more diagnostic tests performed (25.3 *vs.* 17.6, *p* = 0.012) and consumed more medications (3672 *vs.* 2868, *p* = 0.003), as compared to HF patients without DM. In addition, the duration of hospitalisation in HF patients with DM was longer compared to HF without DM (5.8 days *vs.* 4.5 days, *p* = 0.117). Prolonged and more frequent hospitalisation in this group of patients would translate into increased HF’s total healthcare cost (71). Furthermore, previous studies reported that the higher cost of HF in patients with CKD was attributed to the cost of renal replacement therapy (29). However, the present study also found that HF with underlying CKD had significantly longer duration of hospital stay compared to HF without CKD (8.7 days *vs.* 4.5 days, *p* = 0.039). Patients with AF were statistically associated with a higher cost of HF than those without AF because of utilisation of expensive direct oral anticoagulants for thromboembolic event prophylaxis. Conversely, patients with hypertension were associated with a lower cost of HF. Previous cost analysis studies also found the same observation (73, 79). In the present study, the mean procedure cost in patients with hypertension was lower than those without hypertension (USD 497 *vs.* USD 846, *p* = 0.290).

The total healthcare cost of HF increased when NYHA functional class advanced from class I to class IV, with NYHA class IV being the highest cost (9, 42, 43). Similarly in the current study, the median total cost of HF PPPY increased when NYHA advanced from class I to class IV. The substantial increase in the cost of HF was explained by the increased consumption and utilisation of healthcare resources with advanced classes (45). On the contrary, a Polish COI study in HF patients reported that NYHA class I patients had higher healthcare costs than more advanced classes (43). The higher cost of HF in NYHA class I was found to be contributed by expensive intervention procedures aimed at inhibiting disease progression at its early stage (43). Another study which compared the healthcare cost of incident HF against prevalent HF, found that advancing in the NYHA functional classes was not associated with an increase in the cost of HF in the incident group (58). In the current study, although NYHA classes III and IV were associated with a higher cost than less severe classes, the difference is not statistically significant in the multivariate generalised linear model. Patients with advanced NYHA functional class III and IV were not significantly associated with a higher mean cost of HF in the present study could be due to 81.3% of PCI and 81.2% coronary angiogram were performed in patients with NYHA class I and II. The high diagnostic and procedures costs incurred in NYHA classes I and II decreased the cost difference between NYHA classes III and IV.

## Strength and limitations

The strength of this study was that the healthcare cost of HF treatment was estimated using the real-world data and standard COI methodology from patients of three tertiary centres. The results will potentially provide decision-makers and stakeholders with some insights into the economic burden of HF in Malaysia. However, the results in our study need to be interpreted with several limitations. Firstly, this study had inherent limitations associated with retrospective data collection from patients’ medical records. The association of cost and cost predictors were subjected to inaccuracy because not all potential confounding factors were recorded to be included in the analysis. However, data collectors were trained to find deliberately all HF-relevant details from patients’ medical record to increase the accuracy of estimation. Furthermore, expert clinical input was used to identify relevant data for collection.

Secondly, the majority of patients in the current study had been diagnosed with HF for < 1 year (71%). However, usually, the treatment across different pharmacological classes of HF medications is initiated earlier than one year. Thus, the economic burden estimation using these costs may represent an accurate assessment for newly diagnosed patients. Thirdly, this study only captured the healthcare resources that had been utilised in the hospital settings. The costs incurred when patients seek medical treatment at general practitioners or private hospitals and indirect medical costs were not included in the analysis. Hence, the exact economic burden of HF in Malaysia could be underestimated.

## Conclusion

This study estimated the direct cost of HF in tertiary public hospitals in Malaysia. The healthcare cost of HF is driven by inpatient cost, particularly by the cost of procedures and diagnostic tests. Underlying comorbidities further intensify the cost of managing HF. The study’s findings provide vital information on health resource utilisation and expenditure of HF treatment in Malaysia. This will assist decision-makers in prioritising healthcare policy, implementing interventions, and allocating health resources efficiently to improve the management of HF in Malaysia.

## Data availability statement

The original contributions presented in this study are included in the article/Supplementary material, further inquiries can be directed to the corresponding author.

## Ethics statement

The study obtained ethical approval from the Medical Research and Ethics Committee (NMRR-20-2379-56411).

## Author contributions

SCO and WYY were involved in conception and design of the study. JZL performed the formal analysis. SCO and JZL wrote the first draft of the manuscript. All authors contributed to the manuscript revision, read, and approved the submitted version.

## References

[1] Ponikowski P Voors AA Anker SD Bueno H Cleland JGF Coats AJS 2016 ESC guidelines for the diagnosis and treatment of acute and chronic heart failure: the task force for the diagnosis and treatment of acute and chronic heart failure of the European society of cardiology (ESC)developed with the special contribution of the heart failure association (HFA) of the ESC. *Eur Heart J.* (2016) 37:2129–200. 10.1093/eurheartj/ehw128 27206819

[2] Bragazzi NL Zhong W Shu J Abu Much A Lotan D Grupper A Burden of heart failure and underlying causes in 195 countries and territories from 1990 to 2017. *Eur J Prev Cardiol.* (2021) 28:1682–90. 10.1093/eurjpc/zwaa147 33571994

[3] Lee H Oh S-H Cho H Cho H-J Kang H-Y. Prevalence and socio-economic burden of heart failure in an aging society of South Korea. *BMC Cardiovasc Disord.* (2016) 16:215. 10.1186/s12872-016-0404-2 27832754 PMC5103434

[4] Ziaeian B Fonarow GC. Epidemiology and aetiology of heart failure. *Nat Rev Cardiol.* (2016) 13:368–78. 10.1038/nrcardio.2016.25 26935038 PMC4868779

[5] Chong AY Rajaratnam R Hussein NR Lip GY. Heart failure in a multiethnic population in Kuala Lumpur, Malaysia. *Eur J Heart Fail.* (2003) 5:569–74. 10.1016/s1388-9842(03)00013-8 12921820

[6] Braunwald E. The war against heart failure: the lancet lecture. *Lancet.* (2015) 385:812–24. 10.1016/s0140-6736(14)61889-4 25467564

[7] Cook C Cole G Asaria P Jabbour R Francis DP. The annual global economic burden of heart failure. *Int J Cardiol.* (2014) 171:368–76. 10.1016/j.ijcard.2013.12.028 24398230

[8] Liao L Allen LA Whellan DJ. Economic burden of heart failure in the elderly. *PharmacoEconomics.* (2008) 26:447–62. 10.2165/00019053-200826060-00001 18489197

[9] Lesyuk W Kriza C Kolominsky-Rabas P. Cost-of-illness studies in heart failure: a systematic review 2004-2016. *BMC Cardiovasc Disord.* (2018) 18:74. 10.1186/s12872-018-0815-3 29716540 PMC5930493

[10] Shafie AA Tan YP Liau SY Yang SL Liew HB Chaiyakunapruk N. Registry based analysis of cost-of-illness study among stage C heart failure patients at hospital Queen Elizabeth Ii, Sabah, Malaysia. *Health Policy Technol.* (2019) 8:51–60. 10.1016/j.hlpt.2019.01.002

[11] Shafie AA Tan YP Ng CH. Systematic review of economic burden of heart failure. *Heart Fail Rev.* (2018) 23:131–45. 10.1007/s10741-017-9661-0 29124528

[12] Callender T Woodward M Roth G Farzadfar F Lemarie JC Gicquel S Heart failure care in low- and middle-income countries: a systematic review and meta-analysis. *PLoS Med.* (2014) 11:e1001699. 10.1371/journal.pmed.1001699 25117081 PMC4130667

[13] Packer M Anker SD Butler J Filippatos G Pocock SJ Carson P Cardiovascular and renal outcomes with empagliflozin in heart failure. *N Engl J Med.* (2020) 383:1413–24. 10.1056/NEJMoa2022190 32865377

[14] McMurray JJ Packer M Desai AS Gong J Lefkowitz MP Rizkala AR Angiotensin–neprilysin inhibition versus enalapril in heart failure. *N Engl J Med.* (2014) 371:993–1004. 10.1056/NEJMoa1409077 25176015

[15] McMurray JJ Solomon SD Inzucchi SE Køber L Kosiborod MN Martinez FA Dapagliflozin in patients with heart failure and reduced ejection fraction. *N Engl J Med.* (2019) 381:1995–2008. 10.1056/NEJMoa1911303 31535829

[16] Johnston KM Lakzadeh P Donato BMK Szabo SM. Methods of sample size calculation in descriptive retrospective burden of illness studies. *BMC Med Res Methodol.* (2019) 19:9. 10.1186/s12874-018-0657-9 30626343 PMC6325730

[17] Ministry of Health Malaysia. *Fees (Medical), (Cost of Services) Order 2014.* Putrajaya: Malaysia Attorney General’s Chamber (2014).

[18] Luca L Paul O. *Price Setting and Price Regulation in Health Care: Lessons for Advancing Universal Health Coverage.* Geneva: World Health Organization (2019).

[19] Lee KY Ong TK Low EV Liow SY Anchah L Hamzah S Cost of elective percutaneous coronary intervention in malaysia: a Multicentre Cross-Sectional Costing Study. *BMJ Open.* (2017) 7:e014307. 10.1136/bmjopen-2016-014307 28552843 PMC5541416

[20] Department of Statistics Malaysia. *Consumer Price Index Malaysia.* Kuala Lumpur: Department of Statistics Malaysia (2021).

[21] Bank Negara Malaysia. *Exchange Rate [Internet].* (2021). Available online at: https://www.bnm.gov.my/exchange-rates?p_p_id=bnm_exchange_rate_display_portlet&p_p_lifecycle=0&p_p_state=normal&p_p_mode=view&_bnm_exchange_rate_display_portlet_monthStart=0&_bnm_exchange_rate_display_portlet_yearStart=2019&_bnm_exchange_rate_display_portlet_monthEnd=11&_bnm_exchange_rate_display_portlet_yearEnd=2019&_bnm_exchange_rate_display_portlet_sessionTime=1200&_bnm_exchange_rate_display_portlet_rateType=MR&_bnm_exchange_rate_display_portlet_quotation=rm (accesed August 10, 2021).

[22] R Core Team. *R: A Language and Environment for Statistical Computing.* 4.1.1 ed. Vienna: R Foundation for Statistical Computing (2021).

[23] Ling HS Chung BK Chua PF Gan KX Ho WL Ong EYL Acute decompensated heart failure in a non cardiology tertiary referral centre, sarawak general hospital (Sgh-Hf). *BMC Cardiovasc Disord.* (2020) 20:511. 10.1186/s12872-020-01793-7 33287705 PMC7720602

[24] National Heart Association Malaysia. *Malaysia Heart Failure Registry 2021.* Kuala Lumpur: National Heart Association Malaysia (2021).

[25] Ku H Chung WJ Lee HY Yoo BS Choi JO Han SW Healthcare costs for acute hospitalized and chronic heart failure in South Korea: a multi-center retrospective Cohort Study. *Yonsei Med J.* (2017) 58:944–53. 10.3349/ymj.2017.58.5.944 28792137 PMC5552648

[26] Kanaoka K Okayama S Nakai M Sumita Y Nishimura K Kawakami R Hospitalization costs for patients with acute congestive heart failure in Japan. *Circ J.* (2019) 83:1025–31. 10.1253/circj.CJ-18-1212 30918219

[27] Huang J Yin H Zhang M Ni Q Xuan J. Understanding the economic burden of heart failure in china: impact on disease management and resource utilization. *J Med Econ.* (2017) 20:549–53. 10.1080/13696998.2017.1297309 28286996

[28] Givertz MM Yang M Hess GP Zhao B Rai A Butler J. Resource utilization and costs among patients with heart failure with reduced ejection fraction following a worsening heart failure event. *ESC Heart Fail.* (2021) 8:1915–23. 10.1002/ehf2.13155 33689217 PMC8120411

[29] Dunlay SM Shah ND Shi Q Morlan B VanHouten H Long KH Lifetime costs of medical care after heart failure diagnosis. *Circ Cardiovasc Qual Outcomes.* (2011) 4:68–75. 10.1161/circoutcomes.110.957225 21139091 PMC3057895

[30] Santas E Valero E Mollar A García-Blas S Palau P Miñana G Burden of recurrent hospitalizations following an admission for acute heart failure: preserved versus reduced ejection fraction. *Rev Esp Cardiol.* (2017) 70:239–46. 10.1016/j.rec.2016.06.021 27816423

[31] Tromp J Paniagua SMA Lau ES Allen NB Blaha MJ Gansevoort RT Age dependent associations of risk factors with heart failure: pooled population based Cohort Study. *BMJ.* (2021) 372:n461. 10.1136/bmj.n461 33758001 PMC7986583

[32] Li P Zhao H Zhang J Ning Y Tu Y Xu D Similarities and differences between HFmrEF and HFpEF. *Front Cardiovasc Med.* (2021) 8:678614. 10.3389/fcvm.2021.678614 34616777 PMC8488158

[33] Raja Shariff RE Kasim S Borhan MK Yusoff MR. Acute heart failure – the ‘Real’ Malaysian experience: an observational study from a single non-cardiac centre. *Proc Singapore Healthc.* (2021) 30:218–24. 10.1177/2010105820978664

[34] Ministry of Health Malaysia. *National Health and Morbidity Survey (Nhms) 2019: Vol. I: Ncds—Non-Communicable Diseases: Risk Factors and Other Health Problems.* Putrajaya: Institute for Public Health, National Institutes of Health (2020).

[35] Chioncel O Lainscak M Seferovic PM Anker SD Crespo-Leiro MG Harjola V-P Epidemiology and one-year outcomes in patients with chronic heart failure and preserved, mid-range and reduced ejection fraction: an analysis of the ESC heart failure long-term registry. *Eur J Heart Fail.* (2017) 19:1574–85. 10.1002/ejhf.813 28386917

[36] Wang H Li YY Chai K Zhang W Li XL Dong YG Contemporary epidemiology and treatment of hospitalized heart failure patients in real clinical practice in China. *Zhonghua Xin Xue Guan Bing Za Zhi.* (2019) 47:865–74. 10.3760/cma.j.issn.0253-3758.2019.11.004 31744275

[37] Tsuji K Sakata Y Nochioka K Miura M Yamauchi T Onose T Characterization of heart failure patients with mid-range left ventricular ejection fraction-a report from the Chart-2 Study. *Eur J Heart Fail.* (2017) 19:1258–69. 10.1002/ejhf.807 28370829

[38] Yingchoncharoen T Wu T-C Choi D-J Ong TK Liew HB Cho M-C. Economic burden of heart failure in Asian countries with different healthcare systems. *Korean Circ J.* (2021) 51:681–93. 34227265 10.4070/kcj.2021.0029 PMC8326210

[39] Urbich M Globe G Pantiri K Heisen M Bennison C Wirtz HS A systematic review of medical costs associated with heart failure in the USA (2014-2020). *Pharmacoeconomics.* (2020) 38:1219–36. 10.1007/s40273-020-00952-0 32812149 PMC7546989

[40] Corrao G Ghirardi A Ibrahim B Merlino L Maggioni AP. Burden of new hospitalization for heart failure: a population-based investigation from Italy. *Eur J Heart Fail.* (2014) 16:729–36. 10.1002/ejhf.105 24806352

[41] Zugck C Müller A Helms TM Wildau HJ Becks T Hacker J Health economic impact of heart failure: an analysis of the nationwide German database. *Dtsch Med Wochenschr.* (2010) 135:633–8. 10.1055/s-0030-1251912 20333603

[42] Delgado JF Oliva J Llano M Pascual-Figal D Grillo JJ Comín-Colet J Health care and nonhealth care costs in the treatment of patients with symptomatic chronic heart failure in Spain. *Rev Esp Cardiol.* (2014) 67:643–50. 10.1016/j.rec.2013.12.014 25037543

[43] Czech M Opolski G Zdrojewski T Dubiel JS Wizner B Bolisȩga D The costs of heart failure in Poland from the public payer’s perspective. Polish programme assessing diagnostic procedures, treatment and costs in patients with heart failure in randomly selected outpatient clinics and hospitals at different levels of care: Polkard. *Kardiol Pol.* (2013) 71:224–32. 10.5603/kp.2013.0032 23575775

[44] Ogah OS Stewart S Onwujekwe OE Falase AO Adebayo SO Olunuga T Economic burden of heart failure: investigating outpatient and inpatient costs in Abeokuta, Southwest Nigeria. *PLoS One.* (2014) 9:e113032. 10.1371/journal.pone.0113032 25415310 PMC4240551

[45] Alghamdi A Algarni E Balkhi B Altowaijri A Alhossan A. Healthcare expenditures associated with heart failure in Saudi Arabia: a Cost of Illness Study. *Healthcare.* (2021) 9:988. 10.3390/healthcare9080988 34442125 PMC8391138

[46] Tatari S Soubra L Tamim H Akhras K Kabbani S. The economic impact of patients with heart failure on the lebanese healthcare system. *ESC Heart Fail.* (2015) 2:178–83. 10.1002/ehf2.12038 28834672 PMC6410541

[47] Kuwabara K Matsuda S Anan M Fushimi K Ishikawa KB Horiguchi H Difference in resource utilization between patients with acute and chronic heart failure from Japanese administrative database. *Int J Cardiol.* (2010) 141:254–9. 10.1016/j.ijcard.2008.11.197 19157584

[48] Titler MG Jensen GA Dochterman JM Xie XJ Kanak M Reed D Cost of hospital care for older adults with heart failure: medical, pharmaceutical, and nursing costs. *Health Serv Res.* (2008) 43:635–55. 10.1111/j.1475-6773.2007.00789.x 18370971 PMC2442365

[49] Ondrackova B Miklik R Parenica J Spinar J Sticha M Sulcova A. In hospital costs of acute heart failure patients in the czech republic. *Cent Eur J Med.* (2009) 4:483–9. 10.2478/s11536-009-0056-z

[50] Rihova B Parenica J Jarkovsky J Miklik R Sulcova A Littnerova S Cardiology department hospitalization costs in patients with acute heart failure vary according to the etiology of the acute heart failure: data from the ahead core registry 2005–2009. *Cor Vasa.* (2013) 55:e7–14. 10.1016/j.crvasa.2012.10.004

[51] Peters-Klimm F Halmer A Flessa S Szecsenyi J Ose D. What drives the costs of heart failure care in Germany? A health services cost analysis. *J Public Health.* (2012) 20:653–60. 10.1007/s10389-012-0501-3

[52] Parissis J Athanasakis K Farmakis D Boubouchairopoulou N Mareti C Bistola V Determinants of the direct cost of heart failure hospitalization in a public tertiary hospital. *Int J Cardiol.* (2015) 180:46–9. 10.1016/j.ijcard.2014.11.123 25438208

[53] Sözmen K Pekel Ö Yılmaz TS Şahan C Ceylan A Güler E Determinants of inpatient costs of angina pectoris, myocardial infarction, and heart failure in a university hospital setting in Turkey. *Anatol J Cardiol.* (2015) 15:325–33. 10.5152/akd.2014.5320 25413230 PMC5336844

[54] Stålhammar J Stern L Linder R Sherman S Parikh R Ariely R Resource utilization and cost of heart failure associated with reduced ejection fraction in Swedish patients. *J Med Econ.* (2012) 15:938–46. 10.3111/13696998.2012.686464 22510016

[55] Agvall B Borgquist L Foldevi M Dahlström U. Cost of heart failure in Swedish primary healthcare. *Scand J Prim Health Care.* (2005) 23:227–32. 10.1080/02813430500197647 16272071

[56] Vondeling GT Cao Q Postma MJ Rozenbaum MH. The impact of patent expiry on drug prices: a systematic literature review. *Appl Health Econ Health Policy.* (2018) 16:653–60. 10.1007/s40258-018-0406-6 30019138 PMC6132437

[57] Ory C Vanderplas A Dezii C Chang E. Congestive heart failure: attributable costs within the managed care setting. *J Pharmaceut Finance Econ Pol.* (2005) 14:87–97.

[58] Liao L Jollis JG Anstrom KJ Whellan DJ Kitzman DW Aurigemma GP Costs for heart failure with normal vs reduced ejection fraction. *Arch Intern Med.* (2006) 166:112–8. 10.1001/archinte.166.1.112 16401819

[59] Wang G Zhang Z Ayala C Wall HK Fang J. Costs of heart failure-related hospitalizations in patients aged 18 to 64 years. *Am J Manag Care.* (2010) 16:769–76. 20964473

[60] Escobar C Varela L Palacios B Capel M Sicras A Sicras A Costs and healthcare utilisation of patients with heart failure in Spain. *BMC Health Serv Res.* (2020) 20:964. 10.1186/s12913-020-05828-9 33081776 PMC7576860

[61] Olchanski N Vest AR Cohen JT DeNofrio D. Comparing inpatient costs of heart failure admissions for patients with reduced and preserved ejection fraction with or without type 2 diabetes. *Cardiovasc Endocrinol Metab.* (2020) 9:17–23. 10.1097/XCE.0000000000000190 32104787 PMC7041871

[62] Agency for Healthcare Research and Quality. *Guide to Prevention Quality Indicators: Hospital Admission for Ambulatory Care Sensitive Conditions.* (2007). Available online at: https://www.qualityindicators.ahrq.gov/Downloads/Modules/PQI/V31/pqi_guide_v31.pdf (accessed October 1, 2021).

[63] Butler J Yang M Manzi MA Hess GP Patel MJ Rhodes T Clinical course of patients with worsening heart failure with reduced ejection fraction. *J Am Coll Cardiol.* (2019) 73:935–44. 10.1016/j.jacc.2018.11.049 30819362

[64] Krittayaphong R Permsuwan U. Cost-effectiveness analysis of sacubitril-valsartan compared with enalapril in patients with heart failure with reduced ejection fraction in Thailand. *Am J Cardiovasc Drugs.* (2018) 18:405–13. 10.1007/s40256-018-0288-x 29926351

[65] Liang L Bin-Chia Wu D Aziz MIA Wong R Sim D Leong KTG Cost-effectiveness of sacubitril/valsartan versus enalapril in patients with heart failure and reduced ejection fraction. *J Med Econ.* (2018) 21:174–81. 10.1080/13696998.2017.1387119 28959905

[66] Cariou C Duteil E Sion M Mahieu N Duco J Achouba A. Budget impact analysis of sacubitril/valsartan introduction for heart failure treatment from the french hospital perspective. *Value Health.* (2017) 20:A607. 10.1016/j.jval.2017.08.1185

[67] Ouwerkerk W Teng TK Tromp J Tay WT Cleland JG van Veldhuisen DJ Effects of combined renin-angiotensin-aldosterone system inhibitor and beta-blocker treatment on outcomes in heart failure with reduced ejection fraction: insights from Biostat-Chf and Asian-Hf registries. *Eur J Heart Fail.* (2020) 22:1472–82. 10.1002/ejhf.1869 32583922

[68] Chew KS Chan HC. Prehospital care in Malaysia: issues and challenges. *Int Paramedic Pract.* (2011) 1:47–51. 10.12968/ippr.2011.1.2.47

[69] Lim YMF Ong SM Koudstaal S Hwong WY Liew HB Rajadurai J Trends for readmission and mortality after heart failure hospitalisation in Malaysia, 2007 to 2016. *Glob Heart.* (2022) 17:20. 10.5334/gh.1108 35342695 PMC8916062

[70] Othman CN Farooqui M. Traditional and complementary medicine: perspective of patients in Sabah, Malaysia. *J Asian Behav Stud.* (2018) 3:180–90. 10.21834/jabs.v3i10.317

[71] Bogner HR Miller SD de Vries HF Chhatre S Jayadevappa R. Assessment of cost and health resource utilization for elderly patients with heart failure and diabetes mellitus. *J Card Fail.* (2010) 16:454–60. 10.1016/j.cardfail.2010.01.007 20610226 PMC2911028

[72] Olchanski N Vest AR Cohen JT Neumann PJ DeNofrio D. Cost comparison across heart failure patients with reduced and preserved ejection fractions: analyses of inpatient decompensated heart failure admissions. *Int J Cardiol.* (2018) 261:103–8. 10.1016/j.ijcard.2018.03.024 29657034

[73] Ziaeian B Sharma PP Yu TC Johnson KW Fonarow GC. Factors associated with variations in hospital expenditures for acute heart failure in the United States. *Am Heart J.* (2015) 169:282–9.e15. 10.1016/j.ahj.2014.11.007 25641538 PMC4316377

[74] Cavender MA Steg PG Smith SC Jr Eagle K Ohman EM Goto S Impact of diabetes mellitus on hospitalization for heart failure, cardiovascular events, and death: outcomes at 4 years from the reduction of atherothrombosis for continued health (reach) registry. *Circulation.* (2015) 132:923–31. 10.1161/circulationaha.114.014796 26152709

[75] Chaudhry SI McAvay G Chen S Whitson H Newman AB Krumholz HM Risk factors for hospital admission among older persons with newly diagnosed heart failure: findings from the Cardiovascular Health Study. *J Am Coll Cardiol.* (2013) 61:635–42. 10.1016/j.jacc.2012.11.027 23391194 PMC3576871

[76] Dunlay SM Redfield MM Weston SA Therneau TM Hall Long K Shah ND Hospitalizations after heart failure diagnosis a community perspective. *J Am Coll Cardiol.* (2009) 54:1695–702. 10.1016/j.jacc.2009.08.019 19850209 PMC2803107

[77] Lawson CA Jones PW Teece L Dunbar SB Seferovic PM Khunti K Association between type 2 diabetes and all-cause hospitalization and mortality in the UK general heart failure population: stratification by diabetic glycemic control and medication intensification. *JACC Heart Fail.* (2018) 6:18–26. 10.1016/j.jchf.2017.08.020 29032131

[78] Arora S Patel P Lahewala S Patel N Patel NJ Thakore K Etiologies, trends, and predictors of 30-day readmission in patients with heart failure. *Am J Cardiol.* (2017) 119:760–9. 10.1016/j.amjcard.2016.11.022 28109560

[79] Whellan DJ Greiner MA Schulman KA Curtis LH. Costs of inpatient care among medicare beneficiaries with heart failure, 2001 to 2004. *Circulation.* (2010) 3:33–40. 10.1161/CIRCOUTCOMES.109.854760 20123669 PMC2818545

