# Supplementary material for: Cost analysis of chronic heart failure management in Malaysia: A multi-centred retrospective study
Source: Front Cardiovasc Med. 2022 Nov 2;9:971592. doi: 10.3389/fcvm.2022.971592 (PMC9666382; doi:10.3389/fcvm.2022.971592)
Supplement: Supplementary file 1 [file Table_1.DOCX]

**S1 Table. List of diagnostic test and procedures included**

| Arterial blood gas (ABG) |
| --- |
| Creatine kinase-muscle bound (CK-MB) |
| Coagulation profile |
| Coronary angiogram |
| C-reactive protein (CRP) |
| CT cardiac |
| Chest X-ray |
| Doppler US- artery per region |
| Electrocardiogram (ECG) |
| Echocardiography (ECHO) |
| Electroencephalogram (EEG) |
| Erythrocyte sedimentation rate (ESR) |
| Full blood count (FBC) |
| Fasting blood sugar (FBG) |
| Haemoglobin A1c (HbA1c) |
| Hepatitis B surface Antigen |
| Hepatitis C Virus Antibody |
| HIV antigen test |
| Holter |
| Iron, total |
| International normalised ratio (INR) |
| Liver function test (LFT) |
| Fasting lipid test |
| MRI (cardiac) |
| Pacemaker-follow up |
| Percutaneous coronary intervention (PCI) |
| Random blood sugar (RBG) |
| Renal function test (RFT) |
| Stress ECHO (Dobutamine) |
| Stress ECHO (Exercise) |
| Thyroid function test (TFT) |
| Troponin I |
| Urea & electrolyte (BUSE) |
| Urine Full Examination, Microscopic Examination (UFEME) |
| Venous blood gas (VBG) |
