# Supplementary material for: Cost analysis of chronic heart failure management in Malaysia: A multi-centred retrospective study
Source: Front Cardiovasc Med. 2022 Nov 2;9:971592. doi: 10.3389/fcvm.2022.971592 (PMC9666382; doi:10.3389/fcvm.2022.971592)
Supplement: Supplementary file 2 [file Table_2.DOCX]

**S2 Table. List of parenteral medications included**

| Amoxicillin-clavulanate 1.2g |
| --- |
| Ampicillin-sulbactam 1.5g |
| Bumetanide 0.5mg/mL |
| Cefoperazone 1g |
| Cefoperazone-sulbactam 1g |
| Ciprofloxacin 200mg |
| Enoxaparin 20mg |
| Enoxaparin 40mg |
| Enoxaparin 60mg |
| Esmolol HCl 10 mg/mL |
| Esomeprazole 40mg |
| Fondaparinux 2.5mg |
| Furosemide 10mg/mL |
| Insulin Aspart 100 IU/ml |
| Insulin Aspart 30% & Protaminated Insulin Aspart 70% 100 IU/mL |
| Insulin Glargine 300 IU/ml |
| Insulin Isophane (rDNA origin) 100IU/mL |
| Insulin soluble (rDNA origin) 100IU/mL |
| Insulin soluble 30% & insulin isophane 70% 100IU/mL |
| Isosorbide dinitrate 1 mg/mL |
| Labetalol 5mg/ml Injection |
| Metoclopramide 10mg |
| Metronidazole 500mg |
| Midazolam 5mg |
| Morphine 2mg/mL |
| Noradrenaline 1mg/mL |
| Omeprazole 40mg |
| Pantoprazole 40mg |
| Ranitidine 50mg |
| Streptokinase 1,500,000 IU |
| Tenecteplase 50mg |
| Tramadol 50mg |
| Unfractionated heparin 5000 units/mL |
