# Supplementary material for: Cost analysis of chronic heart failure management in Malaysia: A multi-centred retrospective study
Source: Front Cardiovasc Med. 2022 Nov 2;9:971592. doi: 10.3389/fcvm.2022.971592 (PMC9666382; doi:10.3389/fcvm.2022.971592)
Supplement: Supplementary file 3 [file Table_3.docx]

**S3 Table. List of oral medications included**

| **ACEi/ARB/ARNi** | **Antiplatelet** | **Anticoagulant** | **Beta-blocker** | **Calcium channel blocker** | **Loop diuretic** |
| --- | --- | --- | --- | --- | --- |
| Captopril 25mg | Aspirin-glycine 100mg | Apixaban 2.5mg | Bisoprolol 2.5mg | Amlodipine 5mg | Bumetanide 1mg |
| Enalapril 20mg | Aspirin 300mg | Apixaban 5mg | Bisoprolol 5mg | Amlodipine 10mg | Furosemide 40mg |
| Enalapril 5mg | Clopidogrel 75mg | Dabigatran 110mg | Carvedilol 6.25mg | Diltiazem 30mg IR * |  |
| Irbesartan 150mg | Ticagrelor 90mg | Dabigatran 150mg | Carvedilol 25mg | Felodipine 5mg ER |  |
| Losartan 100mg |  | Rivaroxaban 15mg | Metoprolol 100mg | Felodipine 10mg ER |  |
| Losartan 50mg |  | Rivaroxaban 20mg | Propranolol 40mg * | Verapamil 30mg * |  |
| Perindopril 4mg |  | Warfarin 1mg |  |  |  |
| Perindopril 8mg |  | Warfarin 3mg |  |  |  |
| Perindopril/indapamide 4mg/1.25mg |  | Warfarin 5mg |  |  |  |
| Ramipril 5mg |  |  |  |  |  |
| Sacubitril/Valsartan 100mg |  |  |  |  |  |
| Sacubitril/Valsartan 200mg |  |  |  |  |  |
| Sacubitril/Valsartan 50mg |  |  |  |  |  |
| Telmisartan 40mg |  |  |  |  |  |
| Telmisartan 80mg |  |  |  |  |  |
| Valsartan 80mg |  |  |  |  |  |

ER: extended release; IR: immediate release

* These medications were not used in the management of heart failure patients with reduced ejection fraction.

| **MRA** | **Nitrates** | **I*_f_* channel blocker** | **Proton pump inhibitor** | **Lipid lowering agents** | **Others** |
| --- | --- | --- | --- | --- | --- |
| Eplerenone 25mg | Glyceryl trinitrate 0.5mg | Ivabradine 5mg | Esomeprazole 40mg | Atorvastatin 20mg | Alfacalcidol 0.25mcg |
| Spironolactone 25mg | Isosorbide dinitrate 10mg | Ivabradine 7.5mg | Omeprazole 20mg | Atorvastatin 40mg | Allopurinol 300mg |
|  | Isosorbide mononitrate 60mg |  | Pantoprazole 40mg | Ezetimibe 10mg | Alprazolam 0.5mg |
|  |  |  |  | Fenofibrate 145 | Amiodarone 200mg |
|  |  |  |  | Pravastatin 20mg | Ascorbic acid 100mg |
|  |  |  |  | Rosuvastatin 10mg | Bromhexine 8mg |
|  |  |  |  | Simvastatin 10mg | Calcitriol 0.25mcg |
|  |  |  |  | Simvastatin 40mg | Calcium carbonate 500mg |
|  |  |  |  |  | Calcium lactate 300mg |
|  |  |  |  |  | Carbimazole 5mg |
|  |  |  |  |  | Colchicine 0.5mg |
|  |  |  |  |  | Ferrous fumarate 200mg |
|  |  |  |  |  | Folic acid 5mg |
|  |  |  |  |  | Mecobalamin 500mcg |
|  |  |  |  |  | Potassium chloride SR 600mg |
|  |  |  |  |  | Prazosin 2mg |
|  |  |  |  |  | Prednisolone 5mg |
|  |  |  |  |  | Ranitidine 150mg |
|  |  |  |  |  | Thiamine 10mg |
|  |  |  |  |  | Tramadol 50mg |
|  |  |  |  |  | Trimetazidine 20mg |
|  |  |  |  |  | Trimetazidine MR 35mg |
|  |  |  |  |  | Vitamin B Complex |
|  |  |  |  |  | Vitamin B1, B6, B12 |
