# Supplementary material for: Cost analysis of chronic heart failure management in Malaysia: A multi-centred retrospective study
Source: Front Cardiovasc Med. 2022 Nov 2;9:971592. doi: 10.3389/fcvm.2022.971592 (PMC9666382; doi:10.3389/fcvm.2022.971592)
Supplement: Supplementary file 4 [file Table_4.DOCX]

**Table S4: Cost distribution of heart failure patients from different hospitals.**

| **Setting** | **Cost Component** | **PGH, n=138** | | **SH, n=77** | | **HQE2, n=114** | |
| --- | --- | --- | --- | --- | --- | --- | --- |
|  |  | **Cost PPPY, (USD)**  **Mean (SD),**  **Median (quartiles ^a^)** | **% of cost PPPY *^b^*** | **Cost PPPY, (USD)**  **Mean (SD),**  **Median (quartiles ^a^)** | **% of cost PPPY *^b^*** | **Cost PPPY, (USD)**  **Mean (SD),**  **Median (quartiles^a^)** | **% of cost PPPY *^b^*** |
| Outpatient, n=329 | Clinic Visits | 99 (76) | 4.8 | 80 (40) | 2.9 | 156 (77) | 13.5 |
|  |  | 97 (65, 129) |  | 65 (65, 97) |  | 129 (97, 192) |  |
|  | Medications (tablets/capsules/vials/ampoules) | 259 (329) | 12.5 | 240 (292) | 8.6 | 129 (109) | 11.1 |
|  |  | 118 (64, 319) |  | 114 (75, 246) |  | 91 (61, 133) |  |
|  | Diagnostic Tests | 164 (138) | 7.9 | 280 (197) | 10.0 | 115 (144) | 9.9 |
|  |  | 126 (69, 231) |  | 274 (128, 411) |  | 55 (22, 141) |  |
|  | Total | 522 (375) | 25.2 | 600 (408) | 21.4 | 401 (228) | 34.5 |
|  |  | 413 (241, 722) |  | 496 (354, 769) |  | 297 (227, 502) |  |
| Inpatient, n=101 | Hospitalisations (bed-day) | 372 (262) | 18.0 | 633 (736) | 22.6 | 348 (209) | 30.0 |
|  |  | 305 (164, 437) |  | 340 (235,550) |  | 281 (235, 450) |  |
|  | Medications (tablets/capsules/vials/ampoules) | 9 (17) | 0.5 | 89 (200) | 3.2 | 16 (19) | 1.4 |
|  |  | 3 (1, 11) |  | 18 (2, 56) |  | 8 (2, 26) |  |
|  | Diagnostic Tests | 443 (246) | 21.4 | 536 (460) | 19.1 | 292 (150) | 25.1 |
|  |  | 424 (242,570) |  | 414 (182, 716) |  | 286 (167, 377) |  |
|  | Procedures | 726 (1,822) | 35.0 | 942 (1,413) | 33.6 | 104 (551) | 9.0 |
|  |  | 0 (0, 0) |  | 0 (0, 2,915) |  | 0 (0, 0) |  |
|  | Total | 1,550 (1,904) | 74.8 | 2,200 (2,184) | 78.6 | 760 (684) | 65.5 |
|  |  | 731 (567,144) |  | 904 (470, 3,547) |  | 603 (466, 776) |  |
| **Total cost PPPY, (USD)** | |  |  |  |  |  |  |
| **Mean (SD)** | | 2,072 (1,393) | 100 | 2,800 (1,587) | 100 | 1,161 (558) | 100 |
| **Median (quartiles ^a^)** | | 720 (406, 1,271) |  | 599 (411, 1,164) |  | 441 (237, 775) |  |

HQE2: Hospital Queen Elizabeth II; PGH: Penang General Hospital; SH: Serdang Hospital; PPPY: per patient per year; USD: United State dollar; SD: standard deviation

^a^ First quartile and third quartile were used to describe the distribution of the cost because the costs were skewed to the right

^b^ Percentage of cost per patient per year was generated for mean cost only.

USD 1 = RM 4.134
